# α-Arrestins participate in cargo selection for both clathrin-independent and clathrin-mediated endocytosis

**DOI:** 10.1242/jcs.175372

**Published:** 2015-11-15

**Authors:** Derek C. Prosser, Anthony E. Pannunzio, Jeffrey L. Brodsky, Jeremy Thorner, Beverly Wendland, Allyson F. O'Donnell

**Affiliations:** 1 Department of Biology, The Johns Hopkins University, Baltimore, MD 21218, USA; 2 Department of Biological Sciences, University of Pittsburgh, Pittsburgh, PA 15260, USA; 3 Division of Biochemistry, Biophysics and Structural Biology, Department of Molecular and Cell Biology, University of California, Berkeley, CA 94720-3202, USA; 4 Department of Biological Sciences, Duquesne University, Pittsburgh, PA 15282, USA

**Keywords:** Plasma membrane, Internalization, Protein trafficking, Ubiquitin ligase, Yeast

## Abstract

Clathrin-mediated endocytosis (CME) is a well-studied mechanism to internalize plasma membrane proteins; however, to endocytose such cargo, most eukaryotic cells also use alternative clathrin-independent endocytic (CIE) pathways, which are less well characterized. The budding yeast *Saccharomyces cerevisiae*, a widely used model for studying CME, was recently shown to have a CIE pathway that requires the GTPase Rho1, the formin Bni1, and their regulators. Nevertheless, in both yeast and mammalian cells, the mechanisms underlying cargo selection in CME and CIE are only beginning to be understood. For CME in yeast, particular α-arrestins contribute to recognition of specific cargos and promote their ubiquitylation by recruiting the E3 ubiquitin protein ligase Rsp5. Here, we show that the same α-arrestin–cargo pairs promote internalization through the CIE pathway by interacting with CIE components. Notably, neither expression of Rsp5 nor its binding to α-arrestins is required for CIE. Thus, α-arrestins are important for cargo selection in both the CME and CIE pathways, but function by distinct mechanisms in each pathway.

## Abstract (precis)

**Summary:** In yeast, α-arrestins bind the Rho1 GTPase and regulate internalization of selective cargo through the clathrin-independent endocytic pathway.

## INTRODUCTION

Organelle identity, membrane composition and cellular signaling rely on accurate protein and membrane sorting. A dynamic interplay between exocytosis and endocytosis is essential to maintain the composition of the plasma membrane. Selective cargo sorting into Golgi-derived secretory vesicles and endosome-derived recycling vesicles, and subsequent targeting and fusion of these vesicles to the plasma membrane, is crucial for delivery of cargo to the cell surface. By contrast, endocytosis internalizes cargo from the plasma membrane, and is important for nutrient uptake, turnover of damaged proteins, regulation of membrane composition and response to extracellular signals.

Sorting decisions differentiate between proteins that will be internalized and those that will remain at the plasma membrane. Proteins often undergo post-translational modification, including phosphorylation and ubiquitylation, before internalization (Goh and Sorkin, 2013; Marchese and Trejo, 2013; Nikko et al., 2008). Modified proteins are recognized by endocytic adaptors, which link membranes, cargos and the endocytic machinery (Polo et al., 2002; Reider and Wendland, 2011; Shih et al., 2002).

Sorting decisions not only determine which proteins are internalized, they also direct cargo into specific endocytic pathways. Clathrin-mediated endocytosis (CME), in which clathrin forms a stabilizing coat around the vesicle, is the best-characterized endocytic mechanism. However, most eukaryotic cells have multiple clathrin-independent endocytic (CIE) pathways (Mayor et al., 2014). CIE pathways promote internalization through alternative mechanisms or cell surface structures, including lipid-enriched membrane microdomains (i.e. caveolae, CLIC–GEEC pathway), membrane ruffles/protrusions (i.e. phagocytosis and macropinocytosis) or activation of Rho- or Arf-family small GTPases (Lamaze et al., 2001; Radhakrishna et al., 1996; Sabharanjak et al., 2002).

*Saccharomyces cerevisiae* is a widely-used model for CME, and has provided important insights into the composition, dynamics and regulation of CME that are widely conserved (Boettner et al., 2012; Kaksonen et al., 2003, 2005). Despite genetic evidence that additional endocytic routes existed (Chu et al., 1996; Payne et al., 1988), yeast were thought to rely solely on CME; however, recent evidence demonstrated the existence of a yeast CIE pathway that depends on the GTPase Rho1 and the formin Bni1 (Prosser and Wendland, 2012; Prosser et al., 2011). In addition, a CIE pathway was discovered in *Candida albicans* (Epp et al., 2013) and an alternative endocytic route in *S. cerevisiae*, using only a few CME proteins, might function when CME is impaired (Aghamohammadzadeh et al., 2014). Thus, yeasts use multiple internalization pathways, but the mechanisms of cargo selection and sorting, as well as regulation of these ‘alternative’ non-CME pathways remain poorly understood in yeast and mammals.

Here, we define a previously unrecognized role for α-arrestins in CIE cargo selection. *S. cerevisiae* α-arrestins are a family of 14 proteins, classified based on predicted structural similarity with mammalian β-arrestins and with well-established roles in cargo sorting during CME and other trafficking intervals (Alvarez, 2008; Becuwe et al., 2012; Lin et al., 2008). Yeast α-arrestins bind cargo proteins and act as adaptors to recruit the E3 ubiquitin protein ligase Rsp5, which in turn ubiquitylates cargo to stimulate recognition by the CME machinery (Lin et al., 2008; Nikko and Pelham, 2009; Nikko et al., 2008). We show that individual α-arrestins, or sets of α-arrestins, promote internalization of the same cargos by both CME and CIE pathways. Furthermore, phospho-regulation of α-arrestin-mediated cargo trafficking, as observed in CME (O'Donnell et al., 2013), also appears to occur during CIE. Strikingly, whereas internalization through CME requires binding of Rsp5 to α-arrestins, binding is dispensable for cargo uptake by CIE. Instead, α-arrestins regulate cargo selection by binding to components of the CIE machinery. Thus, α-arrestins play mechanistically distinct roles in the CME and CIE pathways in *S. cerevisiae*.

## RESULTS

### Specific α-arrestins interact with components of the CIE pathway

**Fig. 1.**
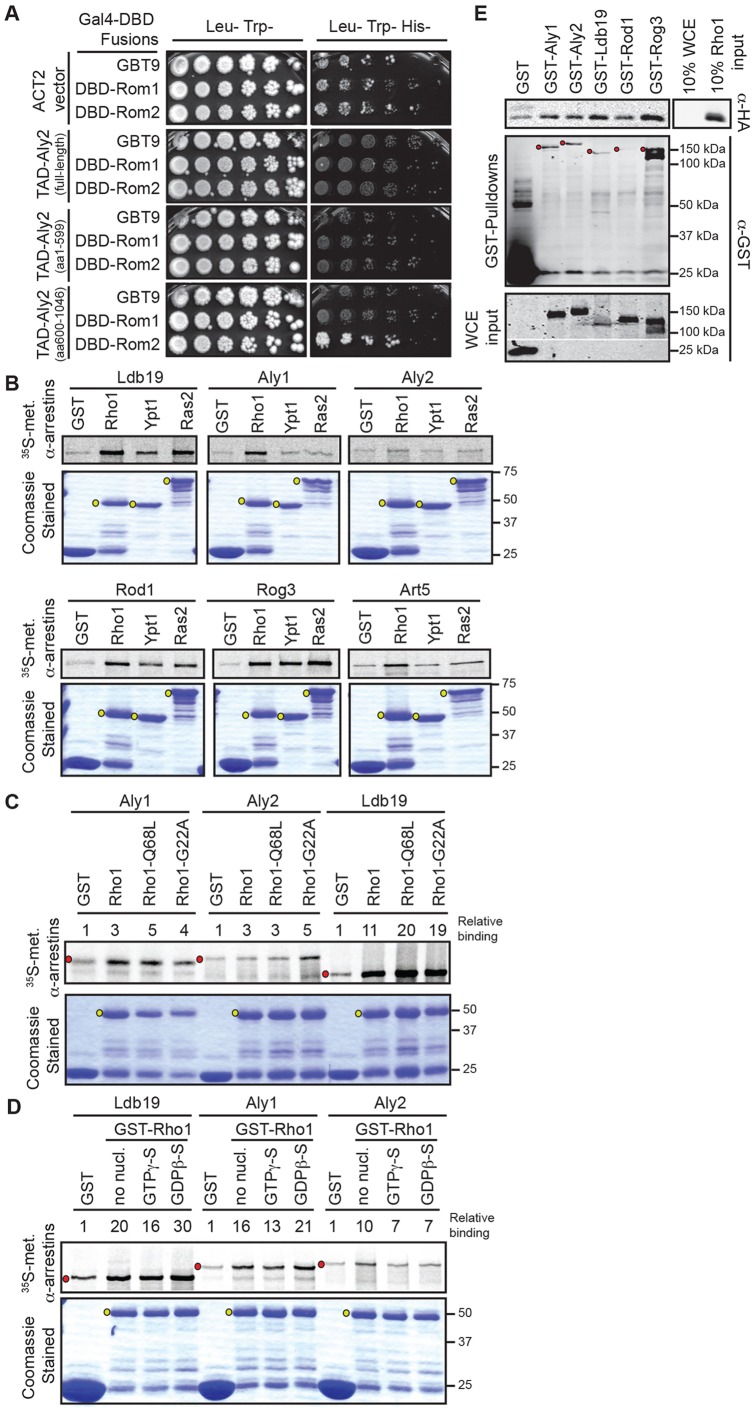
**α-Arrestins interact with the Rho1 GEF Rom2 and the GTPase Rho1.** (A) Yeast two-hybrid analyses of α-arrestin Aly2 fusions to the Gal4 TAD with Rom2 fused to Gal4 DBD. PJ69-4a cells containing the indicated plasmids grown on the indicated media for 4 days at 30°C. (B-D) Purified GST or GST-fused GTPases (Coomassie-Blue-stained gels) incubated with [^35^S]Met-labeled α-arrestins. Co-purifying α-arrestins (top panels) are detected and quantified relative to the amount of GST or GST–GTPase. A representative experiment from at least three replicates is shown. (C) Detection of GST–Rho1, nucleotide-free GST–Rho1^G22A^ or constitutively active GST–Rho1^Q68L^. (D) GST–Rho1 incubated in nucleotide-free buffer or with GTPγS or GDPβS to assess nucleotide specificity of α-arrestins binding to Rho1. (E) Co-purification of HA-Rho1 with GST or GST-α-arrestins extracted from BJ5459 GEV cells assessed by immunoblotting. Red dots indicate full-length α-arrestins; yellow dots indicate full-length GTPase; white lines indicate gel cropping; molecular masses are indicated in kilodaltons.

Previous studies demonstrated a role for the α-arrestins Aly1 and Aly2 in post-endocytic sorting (O'Donnell et al., 2010). To better understand the function of these α-arrestins, we identified potential binding partners by mass spectrometry. Using this approach, two distinct peptides from Rho1 GDP-GTP exchange factor 2 (Rom2) were detected among the proteins that interacted with these α-arrestins. The peptides were present at a 4-fold enrichment with α-arrestin when compared with GST-alone control pull-down experiments (O'Donnell et al., 2010) (A.F.O., A. Apffel, R. G. Gardner and M. S. Cyert, unpublished results). Rom2, and its paralog Rom1, are Rho1-specific guanine nucleotide exchange factors (GEFs) (Ozaki et al., 1996) that contribute to the cell wall integrity pathway (Levin, 2005) and have been implicated in CIE (Prosser et al., 2011). To confirm the association between Aly2 and Rom2, Gal4 DNA-binding domain (DBD) fusions of Rom2 and Rom1 were used as bait in yeast two-hybrid (Y2H) analyses with fusions of the Gal4 transcriptional activation domain (TAD) to Aly2, its N-terminal arrestin domain (residues 1-599) or its C-terminus (residues 600-1046). We found that the Aly2 C-terminus interacted with Rom2, whereas full-length Aly2 did not (Fig. 1A). Exposure of the C-terminus in full-length Aly2 might require some regulatory event. However, we observed previously that full-length TAD–Aly2 is present at lower levels than the TAD–Aly2 C-tail construct (O'Donnell et al., 2013), possibly explaining its lack of interaction with Rom2. Using the same method, TAD fusions to other full-length α-arrestins failed to interact with DBD–Rom1 or DBD–Rom2 (data not shown).

Certain Rho family GTPases bind mammalian β-arrestins (Barnes et al., 2005; Bhattacharya et al., 2002; Claing et al., 2001; Lefkowitz and Shenoy, 2005). Therefore, we explored whether Rho1 can bind to yeast α-arrestins. As judged by Y2H analyses using DBD–Rho1 as bait, robust association with TAD fusions to each of the 14 yeast α-arrestins was not detected (data not shown). As an alternative approach, we examined whether *in vitro* transcribed-translated, radiolabeled α-arrestins associated in pull-down assays with GST–Rho1, GST–Ypt1 (a Rab protein) or GST–Ras2. Only GST–Rho1 consistently retained each of the six α-arrestins tested above the GST control level, and only for Ldb19 and Rog3 was binding to GST–Ras2 comparable to that of GST–Rho1 (Fig. 1B). By using Rho1 mutants (Schmelzle et al., 2002; Sekiya-Kawasaki et al., 2002) locked in the GTP-bound or nucleotide-free state [Rho1^Q68L^ and Rho1^G22A^, respectively (Fig. 1C)] or using non-hydrolysable versions of GTP or GDP (GTPγ-S and GDPβ-S, respectively) (Fig. 1D), we consistently found that binding of the three α-arrestins tested (Ldb19, Aly1 and Aly2) was unaffected. These data suggest that the interface between Rho1 and these α-arrestins does not involve the switch I and switch II regions. We also found that each of the GST–α-arrestins precipitated more HA–Rho1 compared with the GST control when extracts from cells expressing GST or GST–α-arrestin fusions and HA–Rho1 were used. These results suggest that the α-arrestins Aly1, Aly2, Ldb19, Rod1 and Rog3 associate with Rho1 *in vivo* (Fig. 1E).

### α-Arrestins promote cargo internalization in CME-deficient cells

Rho1 is a component of yeast CIE (Prosser and Wendland, 2012; Prosser et al., 2011). Given the observed associations between Aly2 and the Rho1 GEF Rom2 and between α-arrestins and Rho1, we asked whether α-arrestins operate in CIE, as they do in CME (Lin et al., 2008; Nikko and Pelham, 2009). CIE in yeast was identified using a mutant strain (hereafter referred to as 4Δ) lacking four monomeric clathrin-binding adaptor proteins – Ent1 and Ent2 (epsin homologs) and Yap1801 and Yap1802 (AP180/PICALM homologs) (Prosser et al., 2011). *ENT1* and *ENT2* are an essential gene pair; however, expression of the PtdIns(4,5)*P*_2_-binding epsin N-terminal homology (ENTH) domain is sufficient to maintain viability (Aguilar et al., 2006). Although viable, 4Δ cells expressing the ENTH1 domain (4Δ+ENTH1) are defective for CME cargo internalization, whereas additional expression of any one of the four deleted adaptor proteins in 4Δ cells restores CME. In 4Δ+ENTH1 cells, actin patch proteins exhibit grossly aberrant dynamics at the cell cortex and 4Δ+ENTH1 cells are temperature-sensitive at 37°C (Maldonado-Báez et al., 2008; Prosser et al., 2011, 2010).

Dosage suppressors of 4Δ+ENTH1 temperature sensitivity restored growth and improved endocytosis of the plasma membrane proteins Ste3 (a-factor receptor) and Mup1 (methionine permease) (Prosser et al., 2011). Among the candidates identified were plasmids expressing the Rho1-activating cell wall stress sensor Mid2, the Rho1 GEF Rom1 and the GTPase Rho1 (Prosser et al., 2011). The Rom1 paralog Rom2 also suppressed the 4Δ+ENTH1 endocytic defect (D.C.P., unpublished results). The observed suppression required the formin Bni1 and Bni1-binding proteins Spa2 and Bud6, which are subunits of the polarisome complex involved in polarized actin assembly (Sheu et al., 1998); however, suppression was independent of clathrin and other CME machinery (Prosser et al., 2011).

**Fig. 2.**
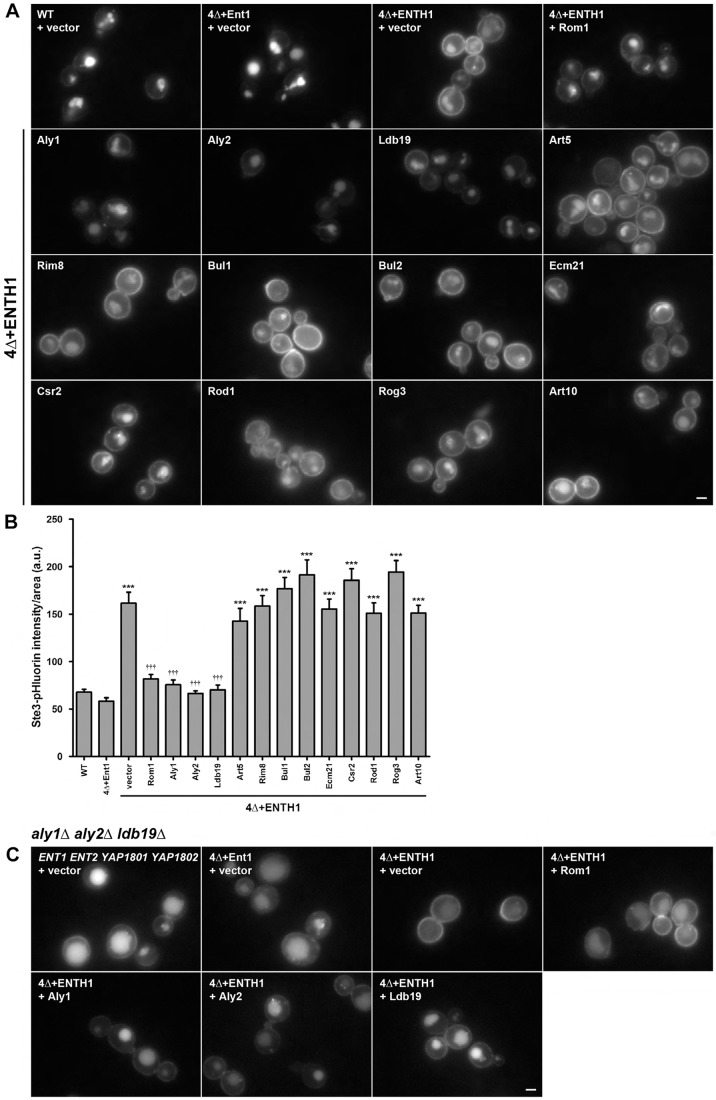
**Overexpression of specific α-arrestins promotes internalization of Ste3–GFP in CME-deficient cells.** (A) WT, 4Δ+Ent1 and 4Δ+ENTH1 cells expressing Ste3–GFP transformed with vector or high-copy plasmids expressing α-arrestins as indicated and imaged by fluorescence microscopy. (B) WT, 4Δ+Ent1 and 4Δ+ENTH1 cells expressing Ste3-pHluorin transformed as in A, and whole-cell fluorescence quantified (arbitrary units, a.u.; ***P<0.001 compared with WT and 4Δ+Ent1 with vector; ^†††^*P*<0.001 compared with 4Δ+ENTH1 with vector). (C) *aly1*Δ *aly2*Δ *ldb19*Δ cells generated in WT, 4Δ+Ent1 and 4Δ+ENTH1 strains expressing Ste3–GFP and transformed with vector or high-copy *ROM1*, *ALY1*, *ALY2*, or *LDB19* plasmids. Scale bars: 2 µm.

On the basis of the published data summarized above, we examined whether high-copy expression of any yeast α-arrestin could suppress the 4Δ+ENTH1 endocytic defect. Ste3–GFP was used to track cargo internalization in these CME-deficient cells because it is constitutively internalized and trafficked to the vacuole in wild-type (WT) cells, but is retained at the plasma membrane when CME is impaired (Maldonado-Báez et al., 2008; Urbanowski and Piper, 2001). As expected, WT cells and 4Δ cells expressing full-length Ent1 (4Δ+Ent1) displayed little plasma membrane fluorescence but pronounced vacuolar fluorescence (Fig. 2A) (Maldonado-Báez et al., 2008; Prosser et al., 2011). By contrast, in 4Δ+ENTH1 cells carrying vector, prominent plasma membrane fluorescence was evident, indicative of defective endocytosis of Ste3–GFP. In 4Δ+ENTH1 cells expressing high-copy Rom1, Ste3–GFP internalization was restored, as evidenced by decreased plasma membrane fluorescence and increased vacuolar fluorescence (Fig. 2A). Likewise, and as judged by the same criterion, high-copy expression of α-arrestins Aly1, Aly2 and Ldb19 promoted internalization of Ste3–GFP, whereas nine other α-arrestins (Art5, Rim8, Bul1, Bul2, Ecm21, Csr2, Rod1, Rog3 and Art10) had little or no effect (Fig. 2A).

The suggestion that Aly1, Aly2 and Ldb19 promote Ste3 internalization by CIE was confirmed by repeating these experiments in cells expressing Ste3 tagged with superecliptic pHluorin, a pH-sensitive GFP variant whose fluorescence is quenched in the acidic vacuole (Miesenböck et al., 1998). This strategy allows the intensity of plasma membrane fluorescence to be quantified in the absence of vacuole-localized signal (Prosser et al., 2010). As seen with Rom1, we found that high-copy expression of Aly1, Aly2 and Ldb19 (but no other α-arrestin) in 4Δ+ENTH1 cells greatly diminished the Ste3–pHluorin signal, to a level comparable to that observed in WT and 4Δ+Ent1 cells (Fig. 2B).

### α-Arrestins promote cargo internalization by CIE

Aly1, Aly2 and Ldb19 could promote Ste3 internalization in 4Δ+ENTH1 cells by several possible mechanisms: by reactivation of CME, through the Rho1-dependent CIE pathway or by another route. To distinguish among these mechanisms, we examined whether high-level Rom1 could still promote Ste3–GFP internalization in 4Δ+ENTH1 cells lacking *ALY1*, *ALY2* and *LDB19*. In cells retaining CME (either the WT or 4Δ+Ent1 background), absence of Aly1, Aly2 and Ldb19 caused a modest retention of Ste3–GFP at the plasma membrane, indicating that these α-arrestins contribute to internalization of Ste3 when CME is intact (Fig. 2C), but also produce a readily detectable vacuolar signal, suggesting that other α-arrestins also contribute to CME of Ste3. Ste3–GFP retention at the plasma membrane was more pronounced in 4Δ+ENTH1 *aly1Δ aly2Δ ldb19Δ* cells than in *aly1Δ aly2Δ ldb19Δ* yeast. Importantly, in these cells, high-level Rom1 expression was impaired in its ability to reduce plasma membrane fluorescence and restore vacuolar localization, whereas high-level expression of any of the three α-arrestins efficiently reduced the plasma membrane fluorescence (Fig. 2C). This result indicates that the Rho1-dependent CIE pathway for Ste3 internalization requires Aly1, Aly2 or Ldb19.

**Fig. 3.**
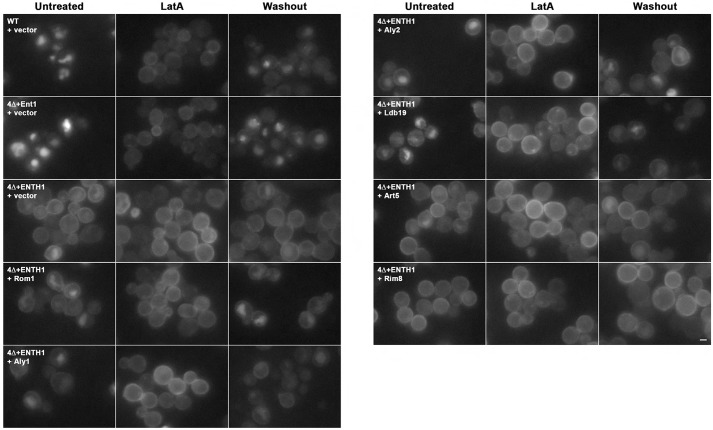
**Latrunculin A treatment to assess requirement for F-actin in Ste3–GFP endocytosis.** WT, 4Δ+Ent1 and 4Δ+ENTH1 cells expressing Ste3–GFP transformed with vector or high-copy plasmids expressing the indicated α-arrestins. Cells were imaged by fluorescence microscopy before (Untreated) or 2 h after (LatA) addition of 200 µM LatA. Following treatment, LatA was washed out, and endocytosis was allowed to resume for 2 h before imaging (Washout). Scale bar: 2 µm.

We next considered the possibility that Aly1, Aly2 and Ldb19 promote vacuole localization of Ste3–GFP in 4Δ+ENTH1 cells by diverting cargo destined for the plasma membrane directly to endosomes or to the vacuole, thwarting Golgi-to-plasma-membrane transport. To address this possibility, we treated cells with the actin-depolymerizing drug latrunculin A (LatA), which blocks endocytosis but not Golgi-to-vacuole transport (Huang and Chang, 2011). After 2 h with LatA, Ste3–GFP accumulated at the plasma membrane in WT and 4Δ+Ent1 cells, consistent with continued plasma membrane delivery and defective endocytosis (Fig. 3). In 4Δ+ENTH1 cells with vector or high-copy Art5 or Rim8, Ste3–GFP was retained at the plasma membrane in untreated cells and showed similar localization after LatA treatment. Importantly, Ste3–GFP accumulated at the plasma membrane in LatA-treated 4Δ+ENTH1 cells with high-copy Rom1, Aly1, Aly2 and Ldb19 (Fig. 3 and Prosser et al., 2011), demonstrating that Rom1 and α-arrestins do not affect transport of cargo to the plasma membrane.

As further evidence for a role for Rom1 and α-arrestins in uptake of plasma membrane cargo, we performed a washout experiment using LatA-treated cells. After accumulation of Ste3–GFP at the plasma membrane, LatA was removed from the medium, allowing resumption of actin polymerization and endocytosis. Under these conditions, Ste3–GFP relocalized from the plasma membrane to the vacuole in WT and 4Δ+Ent1 cells, but remained at the plasma membrane in 4Δ+ENTH1 cells transformed with vector, Art5 or Rim8 (Fig. 3), consistent with impaired endocytosis in CME-deficient cells. By contrast, in 4Δ+ENTH1 cells expressing high-copy Rom1, Aly1, Aly2 and Ldb19, endocytosis of Ste3–GFP was partially restored, as judged by faint, but detectable, vacuolar fluorescence and reduced Ste3–GFP fluorescence at the plasma membrane. Thus, α-arrestins promote endocytosis of plasma membrane cargos (see also later results).

**Fig. 4.**
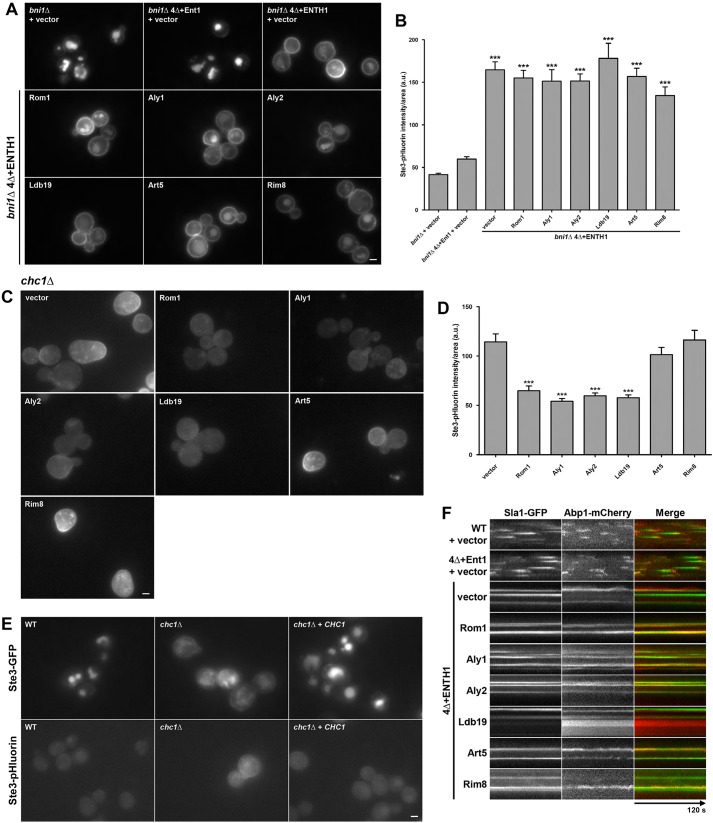
**α-Arrestin-stimulated internalization of Ste3–GFP in CME-deficient cells requires the formin Bni1 but not clathrin and fails to correct cortical actin patch immobility.** (A) *bni1*Δ and WT, 4Δ+Ent1 or 4Δ+ENTH1 strains expressing Ste3–GFP and transformed with vector or the indicated high-copy plasmids examined by fluorescence microscopy. (B) Quantification of fluorescence intensity in *bni1*Δ, *bni1*Δ 4Δ+Ent1 and *bni1*Δ 4Δ+ENTH1 strains expressing Ste3-pHluorin transformed as in A (***P<0.001 compared with *bni1*Δ and *bni1*Δ 4Δ+Ent1 with vector). (C) *chc1*Δ cells expressing Ste3–pHluorin and transformed with vector or the indicated high-copy plasmids examined by fluorescence microscopy. (D) Quantification of fluorescence intensity from cells as shown in C (***P<0.001 compared with chc1Δ with vector). (E) Localization of Ste3–GFP and Ste3–pHluorin in WT and *chc1*Δ cells, as well as in *chc1*Δ cells transformed with a centromeric *CHC1* plasmid. (F) Kymographs derived from time-lapse TIR-FM imaging of WT, 4Δ+Ent1 and 4Δ+ENTH1 cells expressing Sla1–GFP (green) and Abp1–mCherry (red) transformed with the indicated plasmids. Images were captured every second for 120 s. Scale bars: 2 µm.

To verify that α-arrestins act through CIE, we deleted *BNI1* in the 4Δ+ENTH1 cells, as Bni1 is a required component of the CIE pathway (Prosser et al., 2011). As seen previously, the absence of Bni1 did not affect Ste3 internalization in either WT or 4Δ+Ent1 cells, as judged by either Ste3–GFP localization (Fig. 4A) or the Ste3-pHluorin signal intensity (Fig. 4B). In 4Δ+ENTH1 cells lacking *BNI1*, overexpression of Rom1 or any α-arrestin tested was unable to promote efficient Ste3 endocytosis, as judged by the same criteria (Fig. 4A,B). Similar results were obtained in cells lacking the polarisome subunit Spa2 (data not shown), which binds Bni1 (Fujiwara et al., 1998) and is required for CIE (Fujiwara et al., 1998; Prosser et al., 2011). In contrast, in cells lacking another formin, Bnr1, which has no apparent role in CIE (Prosser et al., 2011), Rom1, Aly1, Aly2 or Ldb19 overexpression were effective in stimulating Ste3 endocytosis (Fig. S1A,B). These findings further support the conclusion that Aly1, Aly2 or Ldb19 promote Ste3 internalization by the CIE pathway.

### High-copy α-arrestins promote cargo internalization in a clathrin-independent manner

To determine whether α-arrestins restore endocytosis in 4Δ+ENTH1 cells in a manner that might depend on either clathrin or cortical actin patches, we next assessed the ability of α-arrestins to promote internalization of Ste3 in *chc1Δ* cells, which lack the clathrin heavy chain. Because clathrin functions in vesicle budding from the plasma membrane or from the trans-Golgi network (TGN), *chc1Δ* yeast have defects in multiple trafficking pathways. Furthermore, *chc1Δ* cells have fragmented vacuoles, which can obscure observation of plasma membrane cargo proteins (Burston et al., 2009; Prosser et al., 2011; see also Fig. 4E for a direct comparison of Ste3–GFP and Ste3–pHluorin in WT and *chc1*Δ cells, as well as in *chc1*Δ cells expressing plasmid-borne *CHC1*). Thus, for this analysis, we used *chc1Δ* cells expressing Ste3–pHluorin. As shown in Fig. 4C-E, we observed that surface fluorescence was high in *chc1Δ* cells carrying vector, indicating impaired Ste3–pHluorin internalization. By contrast, surface fluorescence was greatly diminished in cells expressing high-copy *ROM1*, *ALY1*, *ALY2* or *LDB19*, whereas high-copy *ART5* and *RIM8* had no effect. Thus, like Rom1, the α-arrestins Aly1, Aly2 and Ldb19 promote clathrin-independent internalization of Ste3.

To determine whether α-arrestins promote endocytosis in 4Δ+ENTH1 cells without correcting defects in cortical actin patch assembly and dynamics, we performed two-color total internal reflection fluorescence microscopy (TIR-FM) of cells expressing genomically encoded Sla1–GFP and Abp1–mCherry. The timing for recruitment of many yeast actin patch proteins is known, thereby defining discrete stages in CME (Kaksonen et al., 2003, 2005). In brief, an early coat complex forms, which initiates recruitment of clathrin, followed by a late coat complex consisting largely of adaptor proteins and endocytic scaffolds necessary for maturation of the nascent endocytic vesicle. At this time, Arp2/3-mediated actin polymerization and type I myosin (Myo3 and Myo5) activity powers membrane deformation, followed by the action of proteins that sever the neck to separate the vesicle from the plasma membrane. The vesicle then moves toward the cell interior and actin patch proteins dissociate. Arrival of a protein at, and/or its departure from, a cortical patch can be monitored by time-lapse imaging and displayed as a kymograph. Sla1, a late coat protein, arrives at cortical patches 30-40 s prior to completion of endocytosis and is internalized with the vesicle; Abp1, an actin-binding protein, arrives 12-15 s before vesicle internalization (Kaksonen et al., 2005). Thus, Sla1–GFP arrives first (the cortical patch is green), is subsequently joined by Abp1–mCherry (the patch turns yellow), and then both are internalized, which is evident when the patch disappears as the vesicle leaves the TIR-FM field. This stereotypical behavior was readily observed in WT and 4Δ+Ent1 cells (Fig. 4F, top two rows); however, Sla1–GFP patches were static in 4Δ+ENTH1 cells carrying a vector, as seen previously (Prosser et al., 2011). Patches also persisted for over 2 min (the duration of observation) and if a patch became decorated with Abp1–mCherry, it remained similarly static (Fig. 4F). Importantly, high-copy expression of Rom1 or the α-arrestins Aly1, Aly2, Ldb19, Art5 or Rim8 did not alter these patterns (Fig. 4F). Thus, the ability of Aly1, Aly2 and Ldb19 to promote cargo internalization in 4Δ+ENTH1 cells is not mediated by restoration of actin patch dynamics, consistent with its lack of dependence on clathrin heavy chain.

### α-Arrestin function in clathrin-independent endocytosis does not require Rsp5

**Fig. 5.**
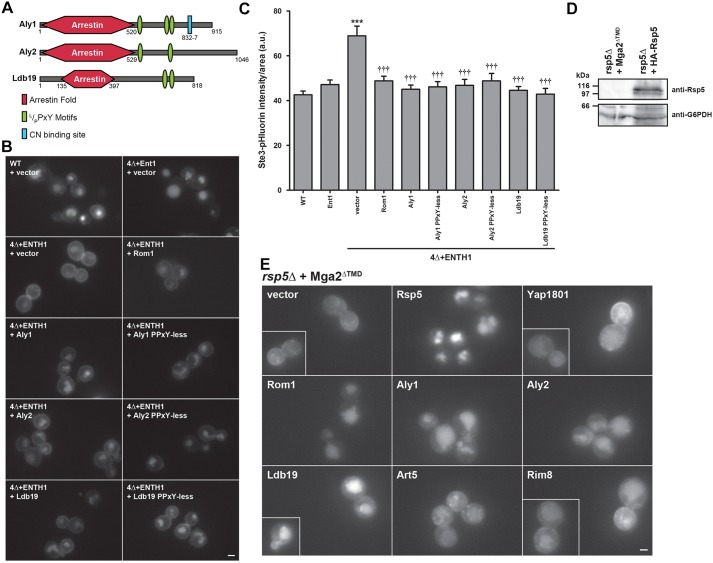
**The Rsp5 ubiquitin ligase is dispensable for α-arrestin-stimulated internalization of Ste3–GFP.** (A) Schematic of α-arrestin primary structure. The arrestin fold (red hexagon) is indicated according to Lin et al. (2008) for Ldb19 and as predicted for Aly1 and Aly2 by Phyre2 (Kelley and Sternberg, 2009; O'Donnell et al., 2010). Green ovals indicate L/PPxY Rsp5-binding motifs and blue rectangle denotes the calcineurin-binding site in Aly1. (B) WT, 4Δ+Ent1 and 4Δ+ENTH1 cells expressing Ste3–GFP and transformed with vector or the indicated high-copy plasmids imaged by fluorescence microscopy. (C) Quantification of fluorescence intensity in WT, 4Δ+Ent1 and 4Δ+ENTH1 cells expressing Ste3–pHluorin transformed as in B (***P<0.001 compared with WT or 4Δ+Ent1 cells with vector; ^†††^*P*<0.001 compared with 4Δ+ENTH1 with vector). (D) Cell extracts from *rsp5*Δ cells expressing plasmid-borne Mga2^ΔTMD^ or HA–Rsp5 resolved by SDS-PAGE and probed with anti-Rsp5 or anti-G6PDH antibodies. (E) *rsp5*Δ cells expressing Mga2^ΔTMD^ and Ste3–GFP transformed with vector or the indicated high-copy plasmids and examined by fluorescence microscopy. Scale bars: 2 µm.

α-Arrestins are characterized by an N-terminal arrestin fold domain involved in cargo recognition and a C-terminal tail containing one or more L/PPxY motifs (Fig. 5A), which bind WW domains in Rsp5 (closest mammalian ortholog is Nedd4L) (Chen and Sudol, 1995; Lin et al., 2008). Thus, α-arrestins serve as adaptors that recruit Rsp5 to cargo, thereby facilitating cargo ubiquitylation. Not surprisingly, α-arrestin–Rsp5 binding is crucial for CME (Lin et al., 2008; Nabhan et al., 2010; Nikko et al., 2008).

To determine whether the function of α-arrestin in CIE similarly requires Rsp5, we first used ‘PPxY-less’ mutants (Alvaro et al., 2014; O'Donnell et al., 2013) in which the consensus L/PPxY motifs of Aly1, Aly2 or Ldb19 were mutated to abolish Rsp5 binding. Unexpectedly, we found that the Aly1, Aly2 and Ldb19 PPxY-less mutants still promoted internalization of Ste3–GFP (Fig. 5B) and Ste3–pHluorin (Fig. 5C) in 4Δ+ENTH1 cells. Moreover, the degree of internalization was indistinguishable from levels observed when the WT α-arrestins were expressed. These data suggest that α-arrestins do not require Rsp5 during CIE, which stands in stark contrast to their function during CME.

One potential caveat to the above conclusion is that the PPxY mutations might weaken but not totally abolish Rsp5 binding (Alvaro et al., 2014). It is also possible that the requirement for Rsp5 during CIE is lower than during CME. To rule out this possibility, we eliminated Rsp5 and repeated these analyses. Normally, Rsp5 is essential because it promotes ubiquitylation and degradation of many proteins. Importantly, Rsp5 ubiquitylates the essential gene pair, *SPT23* and *MGA2* (Hoppe et al., 2000), which leads to proteasomal cleavage of Spt23 and Mga2 at the endoplasmic reticulum (ER) membrane, and releases their cytoplasmic domains to stimulate transcription of oleate synthesis genes (Hoppe et al., 2000; Zhang et al., 1999). Expression of the free cytoplasmic domain of either Spt23 or Mga2 restores viability to *rsp5Δ* cells (Hoppe et al., 2000; Stringer and Piper, 2011), although the cells have a severe endocytic defect.

To examine whether CIE functions in the absence of Rsp5, we constructed an *rsp5Δ* strain expressing the cytoplasmic domain of Mga2 (termed Mga2^ΔTMD^), as well as Ste3–GFP. Immunoblot analysis with Rsp5-specific antibodies confirmed that Rsp5 was absent from *rsp5Δ* cell lysates, but present in lysates from yeast carrying an *RSP5* plasmid (Fig. 5D). In the *rsp5Δ* [Mga2^ΔTMD^] cells carrying vector, Ste3–GFP accumulated at the plasma membrane. Consistent with localization at the plasma membrane, cortically localized Ste3–GFP did not fully overlap with the ER marker RFP–HDEL (Audhya and Emr, 2003) (i.e. some cortical regions contained Ste3–GFP but were devoid of ER membranes), nor was there any perinuclear Ste3–GFP, that would suggest ER retention (Fig. S2). As anticipated, little fluorescent signal was observed in the vacuole, consistent with an endocytic defect. Expression of plasmid-borne *RSP5* greatly reduced surface fluorescence and restored Ste3–GFP trafficking to the vacuole. High-copy *YAP1801*, which functions during ubiquitin-dependent CME, was unable to restore Ste3–GFP internalization, and two high-copy α-arrestins, *ART5* or *RIM8*, were similarly unable to restore Ste3 internalization. Importantly, high-copy expression of *ROM1*, *ALY1*, *ALY2* or *LDB19* reduced Ste3–GFP at the plasma membrane and greatly increased vacuolar fluorescence in *rsp5*Δ+Mga^ΔTMD^ cells. Hence, the three α-arrestins that, when overproduced, promote the CIE of Ste3, still do so in the absence of Rsp5.

### Aly1, Aly2 and Ldb19 downregulate Ste3 and mating pathway signaling in wild-type cells

**Fig. 6.**
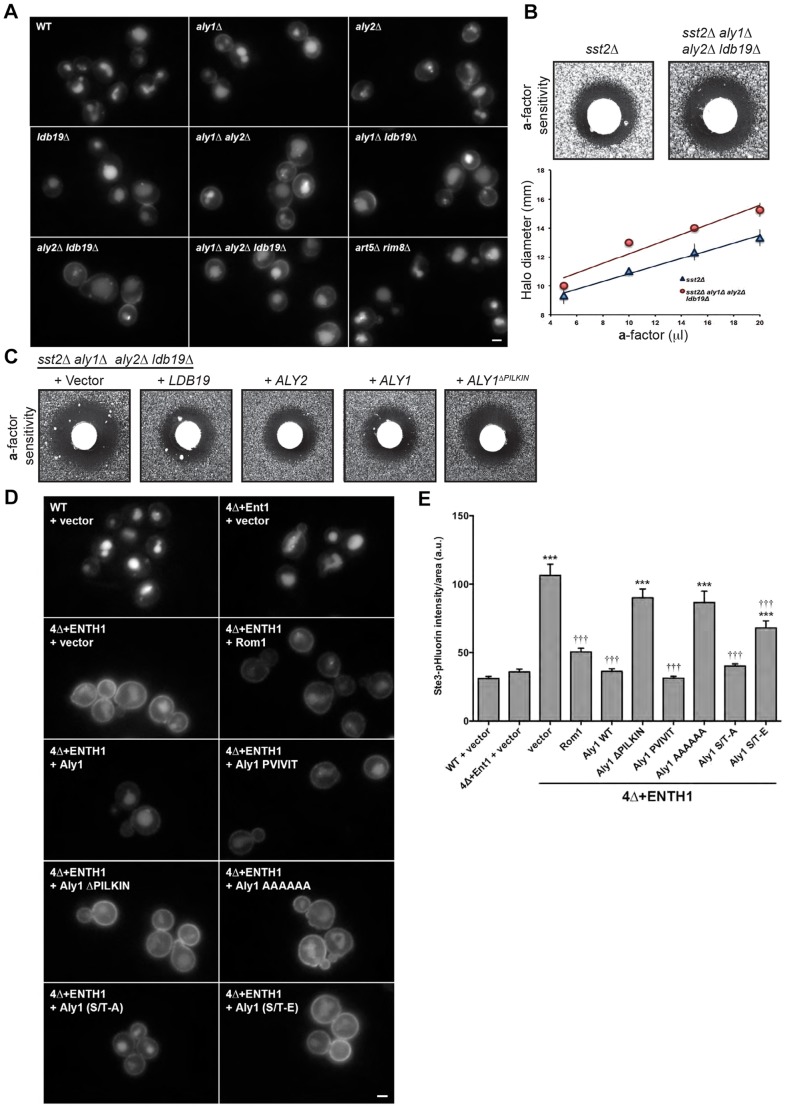
**Aly1, Aly2 and Ldb19 facilitate internalization of Ste3 by CME and the endocytic function of Aly1 requires its calcineurin-mediated dephosphorylation.** (A) Ste3–GFP expressing WT cells and mutant cells bearing the gene deletions indicated examined by fluorescence microscopy. (B) Mating factor-a pheromone sensitivity of cells with the indicated genotype was assessed using an agar diffusion assay for a-factor-induced growth arrest. One of four replicates is shown where 20 µl of a-factor was spotted on the filter disk. The diameter of the zone of growth inhibition was measured across a range of a-factor concentrations for these strains and the diameter of the halo versus a-factor concentration is plotted in the lower panel. Error bars represent the standard deviations (*n*=4). (C) Mating factor-a pheromone sensitivity assays for *sst2*Δ *aly1*Δ *aly2*Δ *ldb19*Δ yeast containing either vector or a centromeric plasmid expressing the indicated α-arrestin allele. (D) WT, 4Δ+Ent1 and 4Δ+ENTH1 cells expressing Ste3–GFP transformed with vector or high-copy *ROM1* or the indicated *ALY1* plasmids examined by fluorescence microscopy. (E) Quantification of fluorescence intensity in WT, 4Δ+Ent1 and 4Δ+ENTH1 cells expressing Ste3–pHluorin transformed as in D (***P<0.001 compared with WT and 4Δ+Ent1 with vector; ^†††^*P*<0.001 compared with 4Δ+ENTH1 with vector). Scale bars: 2 µm.

Although our findings show that Aly1, Aly2 and Ldb19 facilitate Ste3 internalization through CIE, our observations also indicated that these three α-arrestins contribute to Ste3 internalization by CME (Fig. 2C). To corroborate the latter conclusion, we used an independent method to assess whether these three α-arrestins have a discernible impact on Ste3-dependent response to a-factor when CME is intact. High-copy expression of Aly1, Aly2 or Ldb19 stimulated Ste3–GFP internalization in 4Δ+ENTH1 cells, hinting that Aly1, Aly2 and Ldb19 play overlapping roles. For this reason, we generated single (*aly1Δ*, *aly2Δ* and *ldb19Δ*), double (*aly1*Δ* aly2Δ*, *aly1Δ ldb19Δ* and *aly2Δ ldb19Δ*) and isogenic triple (*aly1Δ aly2Δ ldb19Δ*) mutants. Each single mutant exhibited a Ste3–GFP distribution that was indistinguishable from that in WT yeast, indicating efficient receptor internalization (Fig. 6A). However, in cells lacking any pair of these α-arrestins, or all three, Ste3–GFP accumulated at the plasma membrane. In contrast, Ste3–GFP internalization was unimpaired in a strain lacking two other α-arrestins, Art5 and Rim8 (Fig. 6A), which are a paralogous pair analogous to Aly1 and Aly2 and whose individual overexpression had no impact on CIE of Ste3 (Fig. 2). For Aly1, Aly2 and Ldb19, the increased Ste3–GFP plasma membrane fluorescence in cells lacking any two of these α-arrestins suggested that they each help to downregulate the a-factor receptor when CME is intact. When the Ste3 GPCR on the surface of *MAT*α cells binds the a-factor pheromone, the MAP kinase cascade is activated, resulting in changes in gene expression that promote mating and induce growth arrest (Bardwell et al., 1994). Responsiveness to a-factor is assessed by an agar diffusion (‘halo’) bioassay that measures growth arrest of sensitized reporter cells (i.e. *MAT*α *sst2Δ*, where a negative regulator of the mating pathway, an RGS protein, is deleted) that have been challenged with an a-factor-soaked filter disk (Kuchler et al., 1989). We found that *sst2Δ* cells lacking *ALY1*, *ALY2* and *LDB19* were more sensitive to a-factor compared with the *sst2Δ* control cells over a range of concentrations (Fig. 6B). Negative regulation of the mating pathway by these α-arrestins is consistent with their role in internalizing Ste3. It is possible, however, that these α-arrestins contribute in other ways to signal desensitization. Given their ability to both stimulate removal of Ste3 from the plasma membrane and reduce a-factor sensitivity in cells with intact CME, it seems likely that, as in CIE, the same adaptors play a role in internalization of Ste3 by CME. Together these data suggest that, when the efficiency of Ste3 internalization in *MAT*α cells is impaired in the absence of Aly1, Aly2 and Ldb19, greater persistence of Ste3 at the plasma membrane leads to more signaling through the mating pheromone pathway, which, in turn, prolongs growth arrest.

The role for Aly1, Aly2 and Ldb19 in regulating Ste3-dependent response to a-factor is similar to what we recently demonstrated for α-arrestins Rod1, Rog3 and Ldb19 in down-modulating the α-factor receptor (Ste2) in *MAT*a cells (Alvaro et al., 2014). In brief, we found that two paralogous α-arrestins, Rod1 and Rog3, as well as Ldb19 were required for optimal downregulation of the Ste2-dependent mating pathway in response to α-factor and for removal of Ste2 from the plasma membrane. Here, we found a similar requirement for two paralogous α-arrestins, Aly1 and Aly2, as well as Ldb19 in downregulation of Ste3. We next sought to determine whether phospho-regulation of the α-arrestins in these pathways was similarly conserved. Dephosphorylation of Rod1 by calcineurin, which is a phosphoprotein phosphatase, is required for optimal Rod1-mediated negative regulation of Ste2 (Alvaro et al., 2014). Previously, we established that Aly1 is also a calcineurin substrate and that dephosphorylation of Aly1 by calcineurin enhances its ability to mediate removal of a nutrient permease from the plasma membrane (O'Donnell et al., 2013). Therefore, to determine whether calcineurin-dependent dephosphorylation of Aly1 promotes Ste3 endocytosis and negatively regulates Ste3-initiated signaling, we used three approaches. First, we found, as expected, that re-introduction of either Ldb19, Aly2 or Aly1 reduced sensitivity of *MAT*α *aly1Δ aly2Δ ldb19Δ sst2Δ* cells to a-factor-evoked growth arrest, whereas re-introduction of Aly1^ΔPILKIN^, a mutant that no longer binds or is dephosphorylated by calcineurin (O'Donnell et al., 2013), was unable to do so (Fig. 6C). Second, in 4Δ+ENTH1 cells, where overexpression of either Rom1 or WT Aly1 promotes internalization of Ste3–GFP, high-copy Aly1 variants that lack a calcineurin-binding site (Aly1^ΔPILKIN^ and Aly1^AAAAAA^) did not correct Ste3–GFP endocytosis defects, whereas an Aly1 variant with a very-high-affinity calcineurin-binding site (Aly1^PVIVIT^) did (Fig. 6D). Likewise, an Aly1 variant that is permanently dephosphorylated because its calcineurin-regulated phospho-sites (Ser or Thr) have been mutated to Ala, potently stimulated Ste3–GFP endocytosis, whereas an Aly1 variant that mimics the persistently phosphorylated state, because its calcineurin-regulated phospho-sites have been mutated to Glu, did not (Fig. 6D). Third, WT Aly1 and the same set of Aly1 variants that promoted Ste3–GFP internalization also facilitated removal of Ste3–pHluorin from the plasma membrane (Fig. 6E). Taken together, these data indicate that calcineurin-mediated dephosphorylation of Aly1 promotes Ste3 internalization by either the CME or CIE pathways and that particular α-arrestins probably help to select specific endocytic cargo in both the CME and CIE pathways. This model is supported by additional data, provided below.

### α-Arrestins play cargo-selective roles in both the CME and CIE pathways

It is well established that individual α-arrestins bind to and recruit Rsp5 to specific subsets of cargo proteins, promoting their ubiquitylation and CME (Lin et al., 2008). In some cases, multiple arrestins bind to a single cargo (Nikko and Pelham, 2009), as recently shown for interaction of Ldb19, Rod1 and Rog3 with Ste2 (Alvaro et al., 2014). In other cases, a single α-arrestin is mainly responsible for cargo internalization, as demonstrated for Ldb19 and the high-affinity methionine permease (Mup1) (Lin et al., 2008). Because our data suggest that Aly1, Aly2 and Ldb19 promote internalization of Ste3 through both the CME and CIE pathways, we asked whether α-arrestins that act on other cargo do so in both CME and CIE pathways.

**Fig. 7.**
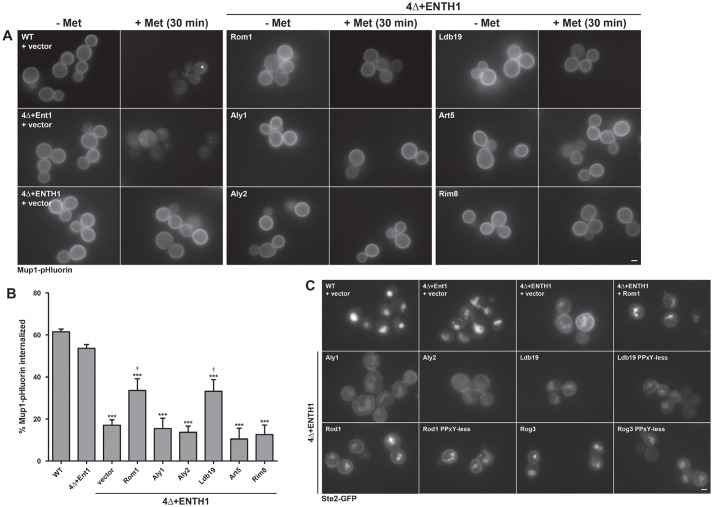
**α-Arrestins have the same cargo-selective roles during both CIE and CME.** (A) WT, 4Δ+Ent1 and 4Δ+ENTH1 cells expressing Mup1-pHluorin transformed with vector or the indicated high-copy plasmids, grown in the absence of methionine imaged by fluorescence microscopy 0 or 30 min after addition of 20 µg/ml methionine. (B) Quantification of fluorescence intensity from experiments shown in A. Values are presented as % internalization after 30 min treatment with methionine (*n*=4; ***P<0.001 compared with WT; ^†^*P*<0.05 compared with 4Δ+ENTH1 with vector). (C) WT, 4Δ+Ent1 and 4Δ+ENTH1 cells expressing Ste2–GFP were transformed with vector or the indicated high-copy plasmids and localization was assessed by fluorescence microscopy. *LDB19*, *ROD1*, *ROG3* and their respective PPxY-less mutants are included. Scale bars: 2 µm.

To examine whether Ldb19 promotes Mup1 internalization by CIE, we monitored internalization of Mup1–pHluorin (Prosser et al., 2010, 2011) in 4Δ+ENTH1 cells. In the absence of methionine, *MUP1* expression is upregulated and the protein is retained at the plasma membrane; in contrast, when excess methionine is present, *MUP1* expression is repressed and the protein traffics to the vacuole and is degraded. Indeed, in WT or 4Δ+Ent1 cells, Mup1–pHluorin was cleared from the plasma membrane 30 min after addition of methionine, whereas little diminution in plasma membrane levels of Mup1–pHluorin was observed in 4Δ+ENTH1 cells (Fig. 7A, left panels). However, in 4Δ+ENTH1 cells, overexpression of Rom1 or Ldb19 (or its L/PPxY variant; data not shown) significantly reduced the fluorescence signal, whereas overexpression of four other α-arrestins did not (Fig. 7A, middle and right panels). These data are summarized and quantified in Fig. 7B. Thus, as occurs during CME, internalization of Mup1 by CIE is promoted by Ldb19.

To address the role of Rod1, Rog3 and Ldb19 during CIE of Ste2, we examined internalization of Ste2–GFP. In *MAT*a cells with intact CME (WT cells and 4Δ+Ent1 cells), in which constitutive internalization of Ste2–GFP is efficient (Alvaro et al., 2014; Schandel and Jenness, 1994), there is barely detectable plasma membrane fluorescence but bright vacuolar fluorescence. In 4Δ+ENTH1 cells, there was readily detectable plasma membrane fluorescence and diminished vacuolar fluorescence (Fig. 7C). Overexpression of *ROM1*, as well as *ROD1* and *ROG3* (and their PPxY-less variants) and to a lesser extent *LDB19* (and its L/PPxY variant), reduced the plasma membrane fluorescence and significantly increased vacuolar fluorescence of Ste2–GFP, whereas overexpression of neither *ALY1* nor *ALY2* did so (Fig. 7C). Therefore, internalization of Ste2 by CIE is promoted by the same set of α-arrestins that are utilized for CME of Ste2.

## DISCUSSION

Cargo sorting maintains the appropriate complement of proteins in each subcellular compartment. Given the array of proteins in a compartment, mechanisms must exist to select and concentrate cargos in nascent vesicles, or to exclude specific proteins from transport sites. The existence of multiple trafficking pathways originating from a single compartment requires cargo packaging into the correct transport intermediate. Thus, cargo sorting must be coordinated to ensure maintenance of organelle identity and cellular function. Many aspects of this process are poorly understood.

**Fig. 8.**
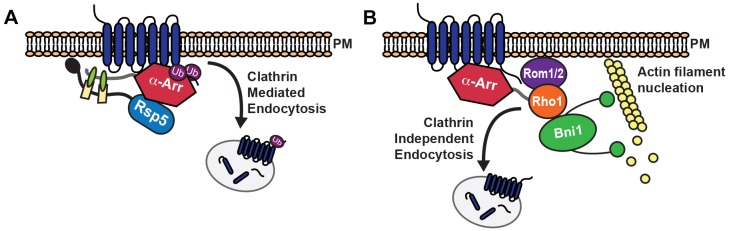
**Schematic depiction of the roles played by α-arrestins as cargo-selective regulators of CME and CIE.** (A) For CME, α-arrestins bind Rsp5 through their L/PPxY motifs and recruit the ligase to cargo proteins. Rsp5 ubiquitylates both the α-arrestin and the cargo to stimulate CME (Alvaro et al., 2014; Lin et al., 2008; Nikko and Pelham, 2009; Nikko et al., 2008; O'Donnell et al., 2013). (B) For CIE, Rsp5 and Rsp5-binding motifs in α-arrestins are dispensable. Instead, we propose that α-arrestins bind cargo proteins and help recruit the Rho1 GTPase and its GEFs, Rom1 or Rom2. Recruitment of Rho1 to the site of cargo internalization stimulates localized activation of the formin Bni1 and subsequent actin nucleation. This model incorporates cargo selection, GTPase activity and actin nucleation, all of which are key features needed to stimulate endocytosis.

On the basis of our findings, we propose that α-arrestins dictate cargo selection and promote cargo internalization through both CME (Fig. 8A) and CIE (Fig. 8B). The former is well established. Specifically, α-arrestins act as adaptors that bind cargo and the ubiquitin ligase Rsp5 to stimulate cargo ubiquitylation (Lin et al., 2008; Nikko and Pelham, 2009; Nikko et al., 2008; Springael et al., 1999). Ubiquitylated cargos are recognized by factors that concentrate endocytic substrates at CME sites (Polo et al., 2002; Shih et al., 2002).

Here, we show that α-arrestins also drive internalization of cargos through CIE. Moreover, we found that α-arrestin function does not require binding or recruitment of Rsp5 in CIE. Instead, our data indicate that α-arrestins interact with components of the CIE pathway, including Rho1 and its GEF Rom2. Thus, α-arrestins are likely to utilize distinct mechanisms to mark cargo for internalization by CIE than they do for CME.

In yeast, there are 14 α-arrestins, each of which contains predicted arrestin-fold domains likely responsible for cargo interaction, and a C-terminal tail with at least one Rsp5-binding L/PPxY motif (Alvarez, 2008; Becuwe et al., 2012). Interaction with cargo and Rsp5 is important for α-arrestin function in CME. The adaptor function of α-arrestins is likely to be conserved. Six mammalian α-arrestins have been identified (five arrestin-domain-containing proteins, ARRDC1–ARRDC5, and thioredoxin-interacting protein, TXNIP) (Alvarez, 2008; Aubry and Klein, 2013). As in yeast, mammalian α-arrestins bind Nedd4-family ubiquitin ligases (Rsp5 homologs) and promote cargo ubiquitylation (Nabhan et al., 2010; Patwari and Lee, 2012; Shea et al., 2012).

A given α-arrestin can bind one or more specific cargos to promote their ubiquitylation and recognition by the CME machinery, thereby contributing to cargo selection and sorting (Alvaro et al., 2014; Becuwe et al., 2012; Lin et al., 2008; Nikko and Pelham, 2009). Conversely, multiple α-arrestins can bind to single cargo and regulate its internalization, indicating that arrestins have partially overlapping functions and/or operate differently under distinct sets of conditions (Alvaro et al., 2014; Nikko and Pelham, 2009; O'Donnell et al., 2015). Indeed, for certain cargos, different α-arrestins promote cargo internalization in response to unique ligands or under different stresses or environmental conditions (Alvaro et al., 2014; Lin et al., 2008; Nikko and Pelham, 2009).

In addition to regulating cargo endocytosis, certain α-arrestins participate in Rsp5-dependent cargo sorting and trafficking at other subcellular compartments. Aly1, Aly2, Bul1 and Bul2 are involved in endosomal sorting of the general amino acid permease Gap1 (O'Donnell et al., 2010; Soetens et al., 2001) and Rod1 contributes to TGN-to-vacuole transport of the lactate/H^+^ symporter Jen1 (Becuwe and Léon, 2014). Moreover, post-translational modifications play key roles in regulating α-arrestin function. Phosphorylation of Ldb19 by the TORC1-inhibited protein kinase Npr1 prevents endocytosis of Ldb19-dependent cargo (MacGurn et al., 2011), whereas Npr1-mediated phosphorylation of Aly2 promotes endosome-to-TGN sorting of Gap1 (O'Donnell et al., 2010). Similarly, phosphorylation of Rod1 by a protein kinase, Snf1, inhibits the endocytic trafficking of Jen1 (Becuwe et al., 2012) and the hexose transporters Hxt1 and Hxt3 (O'Donnell et al., 2015), whereas dephosphorylated Rod1 promotes internalization of Jen1 (Becuwe et al., 2012). Additionally, dephosphorylation of Rod1 and Aly1 by the Ca^2+^/calmodulin-activated phosphatase calcineurin promotes endocytosis of, respectively, the α-factor receptor Ste2 (Alvaro et al., 2014) and the Asp/Glu permease Dip5 (O'Donnell et al., 2013). Also, α-arrestins are themselves substrates for Rsp5-dependent ubiquitylation (Alvaro et al., 2014; Becuwe et al., 2012; Lin et al., 2008; O'Donnell et al., 2013), which may play a role in regulating their function (Becuwe and Léon, 2014; Merhi and André, 2012).

Given the established roles of α-arrestins in CME and in post-endocytic trafficking, how do they contribute to CIE? On the basis of our study, there are some similarities and key differences in how α-arrestins function in CME versus CIE. First, for the cargos tested, selectivity of a given α-arrestin was the same in both pathways: (1) Ste3 internalization involves Aly1, Aly2 and Ldb19; (2) Ste2 internalization involves Rod1, Rog3 and Ldb19; and (3) Mup1 internalization requires Ldb19. Second, as demonstrated for Aly1 in facilitating internalization of Dip5 by CME (O'Donnell et al., 2013), we found that calcineurin-mediated dephosphorylation of Aly1 is necessary to promote internalization of Ste3 by CIE.

In contrast to their CME function, we found that the utilized α-arrestins do not require Rsp5 binding, or even its presence, to stimulate cargo internalization by CIE. Indeed, high-level expression of the CIE component Rom1 or specific α-arrestins promoted Ste3 endocytosis in *rsp5Δ* cells. Initially, our discovery of Rsp5 independence was unexpected, but a previous study indicated that Rsp5 is not strictly required for endocytosis of the Fet3/Ftr1 iron transporter complex *per se*; rather, once internalized, Rsp5-mediated ubiquitylation promotes ESCRT-dependent transport of the complex to the vacuole lumen, effectively preventing its recycling to the plasma membrane (Strochlic et al., 2008). Furthermore, Rog3 does not require Rsp5 binding for its role in downregulation of Ste2 (Alvaro et al., 2014); however, we did not directly assess the ability of Rog3 to promote CME or CIE of Ste2, and so this Rsp5 independence might reflect a role for Rog3 in CIE. Thus, emerging evidence indicates that α-arrestins can play Rsp5-independent roles in trafficking.

Overall, our data suggest that α-arrestins contribute to CIE by serving as molecular matchmakers, but in a fashion that is different from their role in CME. We found that α-arrestins interact with proteins implicated in CIE, including Rom1/2 and Rho1 (Prosser et al., 2011). Similarly, a recent study reported that a fission yeast α-arrestin, Art1, directly associates with the Rho1 GEF Rgf3 (Davidson et al., 2015) and that this interaction is important during cytokinesis; thus, interaction with small GTPases and/or their regulators may be a previously unappreciated but common function of α-arrestins. Indeed, this might apply more globally to the arrestin family, because the mammalian β-arrestins bind several small GTPases, including RhoA (the ortholog of yeast Rho1) and GEFs (Barnes et al., 2005; Bhattacharya et al., 2002; Claing et al., 2001; Lefkowitz and Shenoy, 2005). In this capacity, β-arrestins typically regulate GTPase pathways, similar to the α-arrestin-mediated promotion of Rho1 activity we propose here. It will be interesting in future studies to define the α-arrestin–GTPase binding interface and determine whether this is also conserved across the arrestin family, and to assess the relative contributions of CME and CIE for a specific cargo.

## MATERIALS AND METHODS

### Yeast strains and growth conditions

Yeast strains and plasmids are described in Tables S1 and S2, respectively. Yeast were grown in YPD or SC medium lacking the nutrient(s) required for plasmid maintenance (Alvaro et al., 2014; Prosser et al., 2011) and transformed by the lithium acetate method (Ausubel, 1991).

### Yeast two-hybrid analysis

Y2H tests used PJ69-4a (James et al., 1996) cells containing pGBT9-derived plasmids with Gal4 DBD fusions and pACT2-derived plasmids bearing Gal4 TAD fusions (Bartel et al., 1993; Harper et al., 1993). Transformants were plated on SC-Leu-Trp as a positive control and on SC-Leu-Trp-His to indicate *GAL1_prom_-HIS3* activation.

### *In vitro* binding of α-arrestins to GST–GTPase fusions

BL21 DE3* *E. coli* cells were induced to express GST-tagged Rho1, Ypt1 or Ras2 (pGEX4T-1 plasmids; Table S2) with 100 mM IPTG. After 6 h, cells were collected, washed, resuspended, incubated for 10 min on ice in 1× PBS (pH 7.5) containing 0.2 mg/ml lysozyme, 1 mM DTT, 0.5% Tween-20, 10% glycerol, cOmplete™ mini EDTA-free protease inhibitor tablet (Roche Diagnostics, Mannheim, Germany) and 100 µg/ml DNase and disrupted by sonication. After removal of cell debris, GST-tagged proteins were adsorbed to glutathione-agarose beads for 4 h at 4°C. Beads were washed three times with and resuspended in 20 mM HEPES (pH 7.5), 150 mM NaCl, 5 mM MgCl_2_, 10% glycerol and cOmplete™ mini EDTA-free protease inhibitor. Protein concentration and purity were assessed using the intensity of Coomassie-Blue-stained bands compared with a standard and equivalent amounts of GST, GST–Rho1, GST–Ypt1 and GST–Ras2 were used in each experiment. To examine nucleotide specificity, bead-bound GST–Rho1 was incubated for 30 min with 0.2 mM of GTP, GTPγ-S, GDP or GDPβ-S (Sigma, St Louis, MO) and wash buffers contained 0.2 mM of the corresponding nucleotide.

Radiolabeled α-arrestins, generated as described previously (Alvaro et al., 2014) with the addition of a final purification using Invitrogen Centri-sep Spin Columns (Invitrogen, Carlsbad, CA) to remove residual nucleotides, were incubated with beads containing ∼5 µg of GST-tagged protein at 4°C for 2 h in 20 mM HEPES (pH 7.4), 150 mM NaCl, 5 mM MgCl_2_, 10% glycerol, 0.1% Triton X-100 and cOmplete™ mini EDTA-free protease inhibitor. After two washes with 500 µl of the same buffer, bound protein was eluted in SDS-PAGE sample buffer and resolved by SDS-PAGE. After staining with Coomassie Blue, gels were dried and the level of co-purifying [^35^S]α-arrestins measured using a Phosphorimager screen, a Typhoon scanner and ImageJ software (NIH, Bethesda, MD).

### Co-purification of GST–α-arrestins with HA-tagged Rho1 in cell extracts

BJ5459 GEV cells carrying vector (pEGKG) or plasmids expressing GST–Aly1, GST–Aly2, GST–Ldb19, GST–Rod1 or GST–Rog3 fusions under control of the *GAL10_prom_* were grown and expression induced as described (Alvaro et al., 2014). Cells were lysed by vortexing at 4°C with acid-washed glass beads in 600 µl of 100 mM NaCl, 0.2% Triton X-100, 15 mM EGTA, 50 mM Tris-HCl (pH 7.4) and cOmplete™ mini EDTA-free protease inhibitor. After clarification, GST-tagged proteins were adsorbed to glutathione-agarose beads for 3 h at 4°C. After washing the beads twice in the same buffer, they were incubated at 4°C for 2 h with equal amounts of extract from BJ5459 GEV cells expressing HA-Rho1. After washing three times with 500 µl of the same buffer, bound proteins were eluted in SDS-PAGE sample buffer, resolved by SDS-PAGE and analyzed by immunoblotting using: rabbit polyclonal anti-GST (1:1000 dilution; cat. no. SC-495, Santa Cruz Biotechnologies Inc., Santa Cruz, CA) and mouse monoclonal anti-HA antibody 3F10 (1:1000 dilution; cat. no. 12158167001, Roche Diagnostics). Immune complexes were detected with IRDye (680 or 800)-conjugated anti-rabbit, anti-rat or anti-mouse secondary antibodies (LI-COR Biosciences, Lincoln, NE) and an Odyssey infrared-imaging system (Odyssey™, LI-COR Biosciences).

### Microscopy and image analysis

Image acquisition was performed as described previously for Ste3–GFP, Ste3–pHluorin (Prosser et al., 2010, 2011) and Ste2–GFP (Alvaro et al., 2014). Briefly, cells were grown on selective medium for 16-24 h, dispersed on slides in SC medium and examined at room temperature using an Axiovert 200 inverted microscope (Carl Zeiss, Munich) equipped with a 100×, 1.4 NA Plan-Apochromat objective lens, an X-Cite 120 PC fluorescence illumination system, a Cooke Sensicam (Cooke Corporation, Kelheim) and SlideBook 5 software (3i, Denver, CO).

For TIR-FM imaging of Sla1–GFP and Abp1–mCherry, slides (prepared as above) were viewed at room temperature using a 3i Marianas™ system equipped with a 100×, 1.45 NA Plan-Fluar objective, dual EM charge-coupled device cameras (Cascade II 512, Photometrics, Tucson, AZ), 488 nm and 561 nm diode lasers and SlideBook 5 software. Simultaneous two-color images were acquired at 1 s intervals over 2 min, and kymographs were generated using the multiple kymograph ImageJ plugin (http://www.embl-heidelberg.de/eamnet/html/kymograph.html).

Mup1–pHluorin internalization was tracked as described (Prosser et al., 2011). Briefly, cells grown to mid-exponential phase in SC-Ura-Met were seeded onto concanavalin-A-coated 8-well chamber slides (Nunc) at 30°C. Methionine was added to 20 µg/ml and the cells were viewed immediately and viewed again 30 min later.

LatA treatment and washout experiments were performed using cells grown to mid-exponential phase in SC-Ura. Cells were collected and viewed before or after treatment with 200 µM LatA for 2 h (BioMol, Hamburg, Germany) in SC-Ura at 30°C. Cells were then pelleted at 8000 rpm in a microfuge, washed three times with SC-Ura, and grown for an additional 2 h at 30°C before imaging.

Image acquisition parameters were constant within any experiment, allowing direct comparisons of fluorescence intensities; identical maximum and minimum intensity values were applied using ImageJ. For pHluorin quantification, background was subtracted and whole-cell integrated density values were measured (minimum of 40 cells per condition), normalized to cell size and expressed in arbitrary units (a.u.) for Ste3–pHluorin or as a percentage of initial Mup1 internalized 30 min after methionine exposure. Four independent trials were performed for Mup1 experiments. Significance was assessed by one-way ANOVA, followed by Tukey's multiple comparison analysis.

### Purification of a-factor pheromone and biossay of pheromone-induced growth arrest

The a-factor pheromone was purified as described previously (Sterne, 1989). Briefly, BJ5459 cells (Jones, 1991) containing pKK16 (Kuchler et al., 1989), encoding *MFA1* and *STE6* for robust expression and secretion of a-factor, were grown to saturation in acid-washed glass flasks. The a-factor adheres to glass and was solubilized with n-propanol. Solubilized a-factor was concentrated by rotary evaporation, spotted onto sterile filter disks, and dried. Cellular response to a-factor-impregnated filters was monitored by an agar diffusion bioassay (Kuchler et al., 1989). Approximately 1×10^5^ *MAT*α *sst2Δ* cells were plated in top agar, the disk containing a-factor was placed on the incipient lawn, cells were grown at 30°C for 2-6 days, and the resulting zone of growth inhibition (halo) was measured.
